# Multimodal Personalized Mobile Health Just-in-Time Adaptive Intervention for Occupational Stress Management: Pilot Study

**DOI:** 10.2196/79642

**Published:** 2026-05-18

**Authors:** Beatrix Kunas, Oliver Jung, Christoph Schranz, Mathias Schmoigl-Tonis, Julian Mehlis, Anton-Rupert Laireiter

**Affiliations:** 1 Department of Psychology, University of Salzburg, Hellbrunner Str. 34, Salzburg, 5020, Austria, 43 662 8044-5187; 2 Salzburg Research Forschungsgesellschaft mbH, Salzburg, Austria

**Keywords:** occupational stress, mobile health, mHealth, just-in-time adaptive intervention concept, wearables, personalized interventions

## Abstract

**Background:**

As digital health solutions gain traction, there is an urgent need for effective, person-centered stress management tools for employees. Advances in wearable stress monitoring and machine learning now enable the collection of high-resolution, real-time data and the delivery of personalized interventions with respect to both timing and content. Despite this technological progress, there remains a notable paucity of mobile health (mHealth) interventions that capitalize on these capabilities to implement just-in-time adaptive interventions (JITAIs).

**Objective:**

This pilot study aimed to examine the preliminary effects of the RELAX mHealth app, which uses a JITAI framework to reduce occupational stress, with a particular focus on initial indications of stress reduction, the influence of the app’s degree of personalization on these effects, and overall user experience.

**Methods:**

A 2-arm randomized trial was conducted with 46 employees across two phases of 3 weeks each. Participants used the RELAX mHealth app, which adapts intervention timing based on real-time stress data and tailors content according to users’ preferences. Stress was assessed via multiple heart rate variability (HRV) parameters measured with wearable sensors, alongside subjective questionnaires and ecological momentary assessment (EMA) questionnaires. Usability and user satisfaction were also evaluated. In the second phase, a group randomization was implemented, with two groups of different degrees of in-app personalization.

**Results:**

The findings were mixed. While selected subjective stress indicators decreased from baseline to the end of the study, one physiological measure increased. Retrospective chronic stress (*P*=.048) and arousal related to the most recent stress event prior to EMA entry (*P*=.02) were reduced; however, the low frequency (LF)-to-high frequency (HF) ratio of HRV increased (*P*=.03). Other stress variables, such as work-related stress, valence of daily experiences, and momentary arousal, showed no change over the course of the study. No between-group differences were observed with respect to the assigned degree of personalization. Additionally, usability increased (*P*<.001), whereas satisfaction decreased at a trend level (*P*=.05).

**Conclusions:**

Although the results provide selective preliminary indications of stress reduction with app use, the variability of outcomes raises concerns about its current practical utility. The effects are also discussed with regard to the specific design of the prototype and the study setup. Technical issues and user discomfort were noted despite increasing usability over the study period, indicating areas for improvement in the developed prototype. Given the absence of a control group and the use of interventional arms only, no definitive conclusions about effects can be drawn. Nevertheless, this study highlights both the potential and the challenges of a personalized mHealth approach to occupational stress management. Further refinement of the app and more comprehensive evaluations are needed to fully understand the impact and functional usability of this JITAI-based approach in real-world settings.

## Introduction

### Mobile Health and Individual Occupational Stress

Digital health solutions, including mobile health (mHealth), hold considerable promise as a means of delivering cost-effective, low-threshold interventions to individuals via their personal smartphones [1]. This form of health care has experienced substantial growth in recent years [2], and digital solutions have the potential to deliver effective stress management, particularly for employees [3]. The habitual use of smartphones during working hours [4] presents a significant opportunity for mHealth apps to be used in close proximity to stress-inducing factors, both in terms of temporal and local proximity. However, the precise conditions of occupational stress of the individual employee can vary considerably [5], thus hindering approaches that follow a one-size-fits-all concept. If not fully addressed, individual stress can have a detrimental effect on health and well-being over time [6]. Depending on the individual situation, the consequences range from loss of performance and mental and physical issues [7-9] to economic consequences for the company [10]. It can be concluded that there is a significant need for interventions that optimally address the individual circumstances of employees to prevent the adverse consequences.

Numerous mHealth approaches for occupational stress have been developed across both the unevaluated commercial sector and those with scientifically proven effectiveness [11-13]. The one-size-fits-all approach is predominantly adopted, with a single or limited number of interventions from cognitive behavioral therapy (CBT) being offered in the form of a smartphone app [11-13]. These apps include specific interventions, such as relaxation techniques, psychoeducation, and cognitive methods, with the objective of providing users with generalized low-threshold support in managing occupational stress. The suitability of these apps for specific stress situations is determined by the user, as these apps operate in a passive manner. This highlights the limitations of such apps in providing any depth and personalized approach of traditional psychological counseling or therapy by a qualified professional. The incorporation of individual needs and situational factors, recognized as essential elements in the efficacy of psychological interventions [14], is a crucial aspect that remains beyond the capabilities of these apps.

### Just-in-Time Adaptive Intervention Concept in mHealth

Technical advances in mHealth can replicate traditional face-to-face counseling if they adhere to the just-in-time adaptive intervention (JITAI) concept. JITAI refers to interventions that are delivered at opportune moments and adapt their content to the needs and conditions of the user at those moments [15]. Such an approach requires continuous transmission of real-time personal data to best adapt to prevailing circumstances [16]. This rapidly generates a substantial volume of data, necessitating the incorporation of advanced artificial intelligence (AI) techniques [17]. Algorithms and learning agents, operating discreetly in the background of the app, process the incoming data to ensure optimal personalization [18]. Therefore, the advanced capabilities of mobile devices and the promising concept of JITAIs are mutually beneficial. This approach could multiply personalization by leveraging smartphone technology to provide constant support, potentially surpassing the limitations of conventional, practitioner-based meetings.

In the context of occupational stress, JITAIs have been regarded as a particularly promising medium for providing personalized stress management assistance via smartphones, irrespective of the presence of stress during the workday [19,20]. Design methodologies incorporating personal demographic data to customize the frequency of app interventions are already in practice [21,22]. Nonetheless, these methodologies signify a rudimentary approach to the potential personalization of mobile app use. The development of advanced diagnostic methods for personal stress, such as additional self-report tools and passive sensors, has the potential to enable the app to better understand users’ needs and to adapt in real time for JITAI foundations [23,24]. The use of this multimodal approach in the context of occupational stress is already underway [11,23]. The use of “wearables” is particularly advantageous in this context, as they can be worn during work and thus provide a continuous input of personal stress data for the app without needing much attention [25]. In this respect, heart rate variability (HRV) [26,27] is the stress parameter that is most commonly used and validated.

### Recent Developments and Research

Developments of JITAIs for mHealth are in their infancy, primarily due to the current lack of practical implementations. In general stress management, unobtrusive wearables and app interventions can be effectively combined [28,29], with a few personalized work-stress mHealth offerings identified [23,30]. These approaches often target physical rather than mental stress [31], and most methods limit themselves to monitoring without integrating the data into interventions [24]. This results in the development of personalized apps, but not personalized interventions, thereby not aligning with the JITAI concept. Furthermore, current approaches use either the app or the wearable for measurements and interventions, exhibiting a lack of comprehensive, dynamic integration of multimodal measurement options and data for personalized interventions [23]. mHealth biofeedback apps enable interactive, tailored strategies for occupational stress by integrating real-time sensor data [32]. They provide immediate stress feedback, and when combined with breathing exercises, users can modulate heart rate (HR) through guided breathing, influencing physiological stress [19,33,34]. This interaction supports personalized data analysis and adaptive mHealth interventions. Nevertheless, the time-dependent parameter of the adaptive concept is absent in this instance, thus preventing full compliance with the JITAI concept, leaving users to manage the task of applying the interventions at the optimal time. Moreover, the selection of a singular intervention method restricts the capacity to thoroughly address the user’s unique stress circumstances and individual needs.

While there is one approach that integrates several additional CBT interventions alongside biofeedback for workplace stress, it does not leverage sensor data [19], indicating that the full benefits of ongoing stress monitoring are not fully used. At present, the application to personalize interventions using individual sensor data to tailor the full spectrum of possible interventional conditions, such as content, modules, and presentation timing of the intervention, in line with the JITAI framework, is primarily only conceptualized as such [35,36]. To the best of our knowledge, only one study has formally implemented the concept of automatically selecting CBT interventions based on prior stress indicators measured with a wearable device [37], thereby aligning with the criteria established by JITAI. However, further personalization through long-term learning of individual user preferences, a potential of learning agents, has not been addressed here.

### RELAX Prototype and Research Hypotheses

A prototype based on the JITAI concept was developed and extended with a learning agent to enable longer-term adaptation of interventions within the RELAX project. The prototype is equipped with a smartphone app that gathers both direct and indirect subjective stress data. Concurrently, a wearable wristband sensor is combined to monitor cardiovascular stress indicators. The information gathered from the sensor provides objective measurements of stress levels via HRV. The AI, trained on both personal and stress-related datasets, is responsible for determining the optimal timing for app-based interventions tailored to each user. Additionally, it selects the most appropriate intervention by content for the user at that specific moment, ensuring a personalized approach to stress management. This personalized selection of interventions was preceded by an intensive training phase in which users should evaluate guided access interventions from a pool of a plethora of interventions and thus express their needs and preferences to the AI. At the evaluation time, the training phase was ongoing, but the prototype had achieved the basic JITAI criteria: interventions were triggered by real-time stress, and content could be adaptively selected. More information of the architecture of the app and the personalization process of interventions is described in the study protocol of this study [38].

This study is an analysis of the prototype’s developmental status. It was conducted as a pilot study to test the potential for stress reduction through the app and its feasibility in the everyday work situations of employees. The following research hypotheses were formulated for this purpose:

- Hypothesis 1: A decrease in stress levels in comparison to the participant’s baseline can be observed following the initiation of app use. It is expected that the developed prototype will join the existing effective mHealth applications for stress management. With its structured interventions and diagnostic wearable, it combines proven features [23] and extends them with a JITAI component. The JITAI approach is anticipated to further influence stress by delivering more targeted interventions than prior methods.

- Hypothesis 2: A greater degree of personalization of the app is associated with a greater observed reduction in stress. The benefits of personalization have already been described in previous research on stress management solutions [39], as well as on digital health solutions in general [40], or on behavior change apps in general [41]. Personalization, as the core of the JITAI concept, can have different degrees, thereby giving rise to the question of which nuances are decisive for effectiveness gains. There is evidence of a consistent positive correlation between the degree of personalization and the desired effect [42,43]. However, the exact influence of different forms of personalization is still largely unknown [44].

In addition to the two hypotheses on preliminary app effects, this study assesses the usability of the system. User experience is crucial for mHealth effectiveness [45]. App efficacy hinges on users’ willingness to use it as intended. User concerns, particularly in the domain of health monitoring, encompass a range of issues, including data protection [46], digital stress [47], accessibility, and user control [48]. Prior mHealth work has shown mixed usability: some reported satisfaction [37] and continued use [49], while others faced technical challenges that impaired the user experience [19]. Given these concerns, the study addresses usability and includes supplementary analyses for a broader understanding.

## Methods

### Study Design

The design of this study is described in detail in the corresponding study protocol [38]. The experimental design entailed a 2-arm trial comprising two randomly assigned experimental groups. Participants were required to use the RELAX project app in their naturalistic working environment. The study comprised two phases of 3 weeks each (phase 1 and phase 2). In phase 2, participants were randomly assigned to two distinct levels of personalization of the app (*high* vs *low*). Time series or continuous measurements were obtained via the app and the sensor in the study phases as well as in study-related surveys before the study (T1), between the study phases (T2), and after the study (T3). During the study phases, the participants had access to personalized interventions in the app.

Recruitment was carried out as described in a study by Kunas et al [38]. The ad hoc sample was recruited through previously committed companies and by social media and public flyers. Employees who met the inclusion criteria were given access to the study infrastructure. Adherence during the study was supported by regular reminders via email, the study website, and messages on the app dashboard. Randomization to the experimental groups from phase 2 onward was blinded, as was the evaluation. The allocation of participants and the evaluation process were conducted by different parts of the study team. The participants were randomly assigned to the groups using a pseudo-random function implemented with the NumPy package in the Python programming language. Following the conclusion of the study, participants were given access to the alternative version of personalization.

### Ethical Considerations

The study was ethically approved (EK-GZ 25/2023) and preregistered prior to data analysis [50]. Registration was completed after data collection because modifications to the study design and the prototype were anticipated as the study progressed. The final protocol provides a detailed account of the study design and the prototype architecture and documents all changes, including, for example, the app update implemented in the second week to resolve technical issues identified during the first week. Participants gave their informed consent and were informed that they could opt out of participation at any time without facing any negative consequences. Their consent covered data analyses, especially after anonymization, including secondary analyses. The collected data were pseudonymized with a code for the duration of the study for the purpose of facilitating the identification of the individual participant in the event of technical issues and for the purpose of determining the compensation. Following the completion of the study, all data were anonymized. Upon completion of the study, participants were remunerated with a financial incentive of €80 (US $93.7). Additionally, they were permitted to retain the study sensor at their discretion.

### Intervention

**Figure 1.**
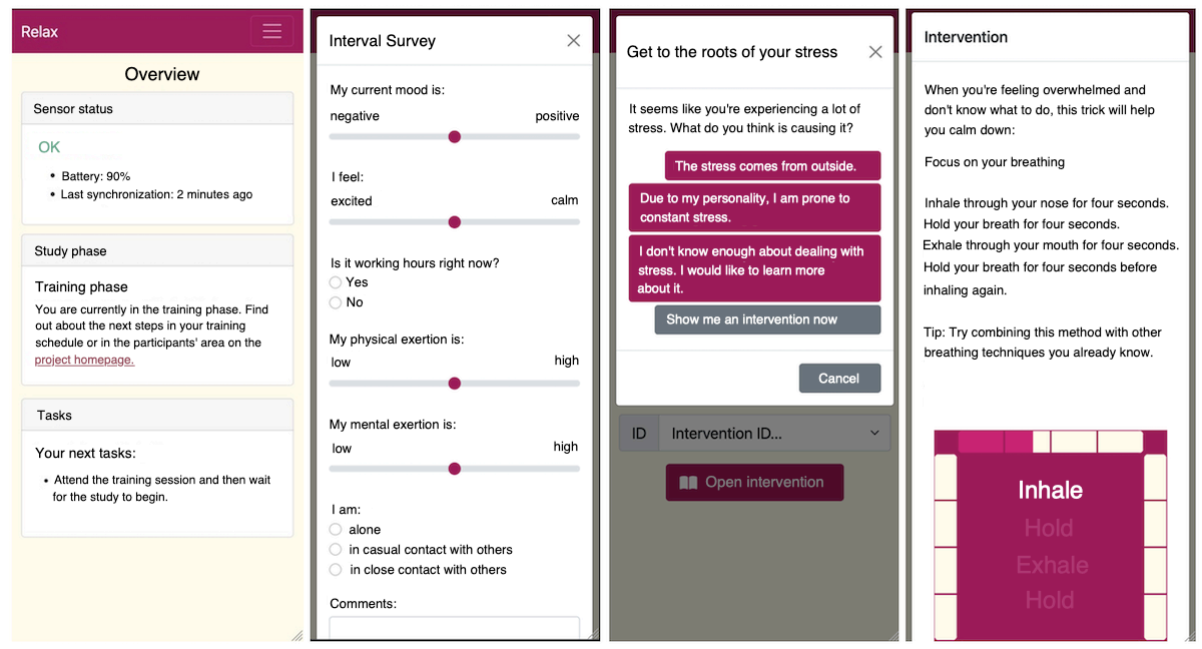
User interface of the RELAX app in English translation, from left to right: dashboard, ecological momentary assessment questionnaire, first level of decision tree, and guided breathing intervention.

The intervention involved the use of the RELAX app in conjunction with the associated sensor. Further details of the app are described in the study protocol [38]. Figure 1 illustrates the user interface of the app in the English translation; the German original of the screen texts is available in the online supplementary materials in Multimedia Appendix 1.

Participants were able to select the self-report questionnaires via the app. The questionnaires had to be completed on a daily basis in the morning and evening in both study phases, as well as several times during the day. The data from the questionnaires served as a measurement tool for the outcomes of the study and as input for the learning agent in the background of the app. In instances where the learning agent identified elevated levels of stress, automated access to a decision tree was initiated, culminating in the provision of suitable microinterventions. In the context of the physiological stress event, personalized current threshold value was calculated for the user (top 25% of values for the quotient of low frequency (LF) vs high frequency (HF), but at least 1.2; bottom 25% of values for the square root of squared mean value of the sum of the differences of successive R-R intervals, but maximum 50 ms; see [38] for detailed description of how the learning agent operated). In the event that both values exceeded or fell below the threshold values in two consecutive 5-minute windows, a stress event was annotated. The subjective triggers remained unaffected by personalization, and the threshold was on the valence scales only, with the mood being ≤3 on the Likert scale (1=negative and 7=positive).

For each branch of the decision tree, the app could recommend several microinterventions from a pool of 179 possible microinterventions for stress reduction. As described in the study protocol [38], the areas of microinterventions included several subareas of CBT, such as psychoeducation, training, or relaxation, which varied in the presentation format. Preset weightings indicated a probability of which microintervention would be triggered in an individual case (see [38] for the distribution of initial weightings). These weightings could be influenced by the user giving feedback ratings after completing the microintervention, which allowed the overall app experience to be personalized over the period of use.

In phase 2, the versions of the app in both experimental groups differed in terms of the extent to which feedback ratings for the microinterventions received were incorporated. The version with *high* personalization modified the weighting of the probabilities for the occurrence of the individual microintervention according to the individual ratings. In contrast, the version with *low* personalization was influenced by the aggregate ratings of the entire group of participants, indicating that no personalization per se was facilitated by the individual user. In phase 1, all participants received the same form of personalization according to the *low* group.

**Figure 2.**
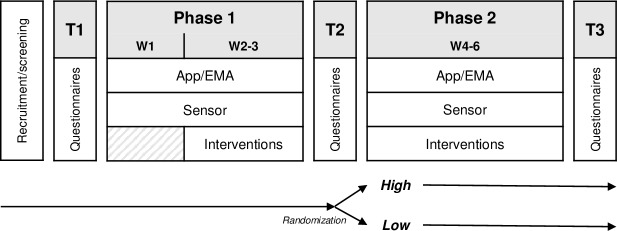
Timeline of the study. EMA: ecological momentary assessment.

The sensor used in this study was the Polar Verity Sense, a wristband that uses photoplethysmography to measure various HRV parameters. Participants were required to wear the sensor continuously throughout the two study phases, with the exception of a 1-hour break each day. The passive nature of the recording process needed minimal involvement on the part of the participants, who were only required to ensure that the sensor was correctly connected to the app. The sequence of events in the study is presented in Figure 2, with the six study weeks abbreviated as W1-6.

### Outcomes

The demographic data were collected at the stage of screening for recruitment prior to the initiation of the study. The main outcomes evaluated in this study are derived from the primary outcomes of the corresponding study protocol (see Kunas et al [38] for measurement times and instruments). The collection of outcome data was facilitated by the use of standardized questionnaires and items developed by the study group via the app and online surveys for subjective data and the sensor for physiological data.

First, stress was measured in chronic form using the items of the daily ecological momentary assessment (EMA), which was created by the study team, as well as in work-related form using the standardized Valencia Eustress-Distress Appraisal Scale (VEDAS) [51] before the start of the study, between the study phases, and after the end of the study. A distinction was made between work-related distress and eustress. Stress was also measured using HRV parameters. These included the SD of R-R intervals, square root of squared mean value of the sum of the differences of successive R-R intervals, and LF/HF, which were measured continuously via the sensor. Further subjective variables encompassed the emotional states of arousal and valence, recorded at the time of entry and regarding the most recent stress event via EMA. Finally, satisfaction with the user experience and the usability of the system were measured using the standardized self-assessment Customer Satisfaction Score [52] and the System Usability Scale [53].

### Statistical Analysis

The data were preprocessed according to the study protocol [38], including generating weekly data for the time series stress data. Datasets with more than 75% missing values were excluded from the analysis, and the procedure was therefore stricter than initially anticipated [38]. To measure the effect of the intervention on stress reduction, pre-post analyses were conducted in a within-subject design with the stress-related and emotional outcomes. Usability and satisfaction were compared separately between T2 and T3. For the between-subject analysis, the *high* versus *low* groups were included in all analyses. The System Usability Scale scores were converted to the standard 0 to 100 scale.

Corresponding linear mixed models (LMMs) were calculated for the analyses, with measurement times and groups as fixed factors and stress levels and usability variables, as measured by the different instruments or indicators, as dependent variables. Post hoc tests with Bonferroni correction were included for the detailed differentiation between measurement times. An α of .05 was used as the significance level, and partial eta-squared (η²*_p_*) indicated the effect size for the LMMs. To control for the false discovery rate, the Benjamini-Hochberg correction was applied. Only the adjusted *P* values are reported.

## Results

### Sample Characteristics

A total of 46 employees participated in the study after four people dropped out in the first 2 weeks of the study. The sample size exceeded the target of 31 participants, as determined by the a priori power analysis for feasibility estimation [38]. The sample consisted of 25 women (54%), 20 men (44%), and 1 diverse person (2%), with an average age of 40 (SD 9.71, range 24-62) years. The majority of the participants were Austrians (n=36, 78%), and the rest were Germans (n=10, 22%). In terms of occupation, the service sector was most strongly represented in the sample with 17 employees (37%), followed by society, sport, or cultural sectors with 8 employees (17%) and the industrial sector with 6 employees (13%). In terms of educational level, the majority of respondents (n=20, 44%) had a postgraduate degree, followed by a university degree (20%) and a high school degree with 9 people each (20%).

**Figure 3.**
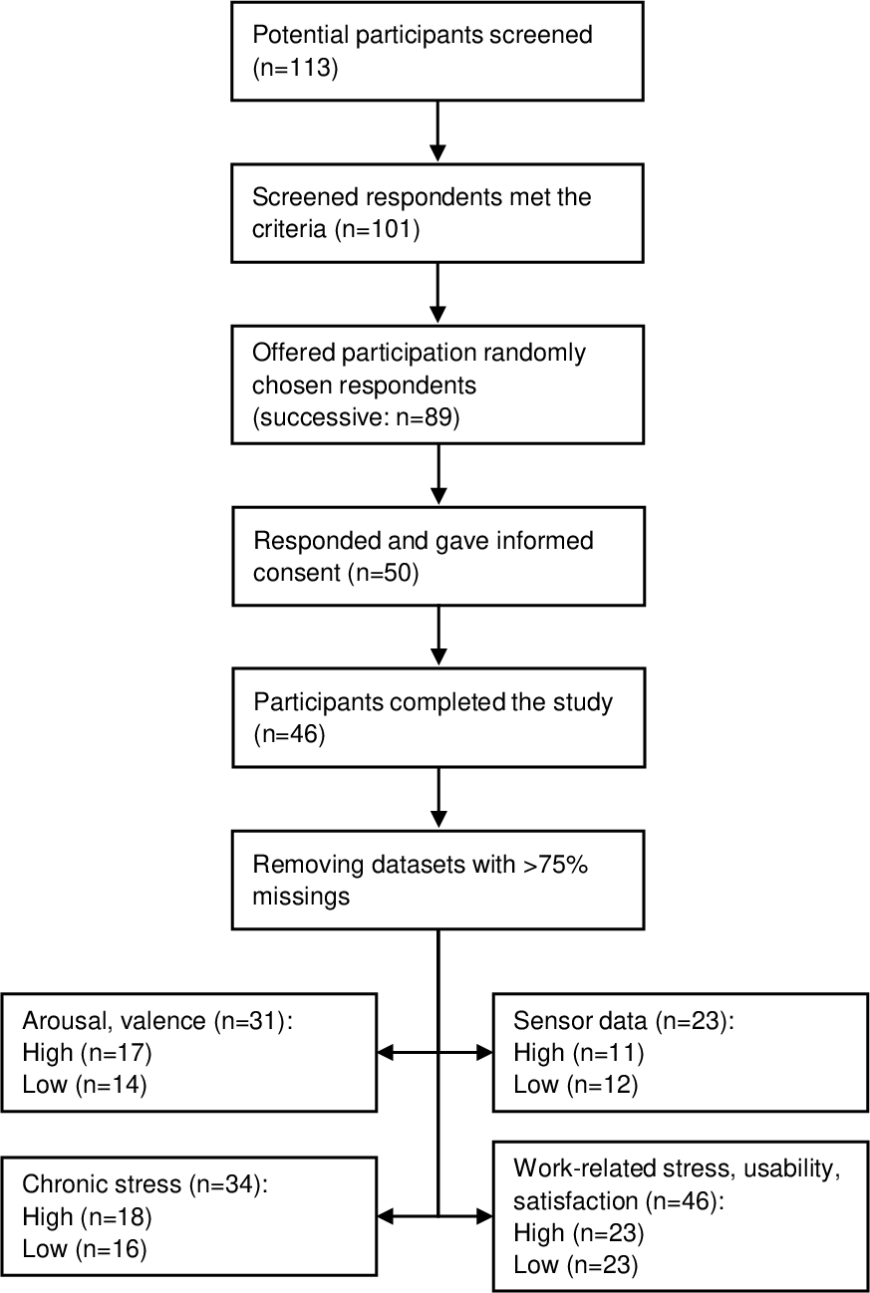
PRISMA (Preferred Reporting Items for Systematic Reviews and Meta-Analyses) flow diagram of the study.

On average, participants received 10.24 interventions (SD 7.41). There was no difference in the number of interventions in phase 2 between the groups (*t*_^44^_=−1.24; *P*=.22). The sample sizes of the individual LMM analyses varied due to missing values, a consequence of the quality criterion on the collected data. This resulted in sample sizes ranging from 22 to 46 in the analyses. The proportion of data from the subjective questionnaires relative to the total possible data varied, on average, between 85% and 100%, depending on the study phase and parameters, and between 45% and 60% for the sensor data. Figure 3 shows the study’s flow diagram, and Table 1 displays the means and SDs of the analyzed outcomes, arranged by time point or study group.

**Table 1.** Study variables.

Variable	Time of measurement, mean (SD)									Groups, mean (SD)	
T1^a^	W1^b^	W2^b^	W3^b^	T2^a^	W4^b^	W5^b^	W6^b^	T3^a^	High	Low
Work-related distress	3.61 (0.95)	—^c^	—	—	3.64 (0.78)	—	—	—	3.74 (0.65)	3.72 (0.72)	3.60 (0.87)
Work-related eustress	3.18 (0.68)	—	—	—	3.17 (0.74)	—	—	—	3.23 (0.71)	3.26 (0.74)	3.13 (0.69)
Daily chronic stress	—	3.13 (0.54)	3.07 (0.57)	3.03 (0.63)	—	3.10 (0.60)	2.90 (0.60)	2.92 (0.56)	—	3.13 (0.63)	2.91 (0.51)
Arousal (momentary)	—	3.33 (0.89)	3.29 (0.77)	3.30 (1.02)	—	3.15 (0.99)	3.12 (1.12)	3.17 (1.06)	—	3.27 (1.03)	3.18 (0.92)
Valence (momentary)	—	5.40 (0.72)	5.23 (0.68)	5.45 (0.85)	—	5.23 (0.91)	5.39 (0.89)	5.45 (0.79)	—	5.13 (0.77)	5.41 (0.87)
Arousal (most recent stress event)	—	4.38 (0.68)	4.37 (0.59)	4.28 (0.85)	—	4.13 (0.63)	4.05 (0.65)	4.03 (0.40)	—	4.23 (0.76)	4.17 (0.43)
Valence (most recent stress event)	—	4.14 (0.61)	4.06 (0.45)	4.32 (0.64)	—	4.07 (0.55)	4.05 (0.52)	4.06 (0.49)	—	4.16 (0.67)	4.07 (0.31)
SDRR^d^	—	19.90 (5.02)	19.18 (4.34)	20.62 (4.38)	—	19.03 (5.15)	19.45 (4.16)	19.41 (4.14)	—	18.66 (4.31)	20.46 (4.70)
RMSSD^e^	—	21.12 (5.60)	20.34 (4.76)	22.01 (4.81)	—	19.99 (5.74)	20.73 (4.81)	20.59 (4.44)	—	19.98 (4.66)	21.54 (5.37)
LF/HF^f^	—	1.52 (0.24)	1.60 (0.43)	1.58 (0.32)	—	1.69 (0.48)	1.65 (0.52)	1.80 (0.60)	—	1.67 (0.46)	1.61 (0.41)
Usability	—	—	—	—	37.35 (8.97)	—	—	—	46.85 (9.21)	43.26 (8.75)	41.03 (9.39)
Satisfaction	—	—	—	—	2.57 (0.64)	—	—	—	2.38 (0.76)	2.66 (0.68)	2.28 (0.68)

a T1-3: measurement times.

b W1-6: study weeks.

c Not applicable.

d SDRR: SD of R-R intervals.

e RMSSD: square root of squared mean value of the sum of the differences of successive R-R intervals.

f LF/HF: quotient of low versus high frequency.

### Stress Reduction (H_1_)

The overall picture reveals only isolated significant results. There was a decrease in daily chronic stress over the course of the study (*F*_5,165_=2.89; *P*=.048; η²*_p_*=0.081). Post hoc analyses revealed no significant differences between weeks; however, there was a tendency between W1 and W5 (*P*=.06). Furthermore, there was a decline in the self-reported arousal of the most recent stress event, as reported from the time of app entry, that was identified over the course of the study period (*F*_5,150_=4.52; *P*=.02; η²*_p_*=0.131). Post hoc tests revealed several effects between the weeks: Specifically, these included comparisons between W1 and W5 (*P*=.02), W1 and W6 (*P*=.02), W2 and W5 (*P*=.04), and W2 and W6 (*P*=.03). However, no significant effects were observed in other subjective parameters, such as work-related stress experience or valence.

The sensor data revealed that only one parameter exhibited significant effects during the study period, with LF/HF increasing over the weeks (*F*_5,109.01_=3.91; *P*=.03; η²*_p_*=0.152). According to the definition of the parameter, this can be interpreted as an increase in sympathetic activity, which indicates increased physiological stress. However, none of the other HRV parameters exhibited a similar pattern. The results of the LMMs are displayed in Table 2.

**Table 2.** Linear mixed models of the study variables.

Variable	Within-subjects analysis			Between-subjects analysis		
	*F* (df)	*P* value	η²_*p*_ (95% CI)	*F* (df)	*P* value	η²_*p*_ (95% CI)
Work-related distress	0.77 (2, 90)	.696	0.017 (0.000-0.085)	0.37 (1, 44)	.73	0.008 (0.000-0.124)
Work-related eustress	0.22 (2, 90)	.80	0.005 (0.000-0.047)	0.59 (1, 44)	.67	0.013 (0.000-0.139)
Daily chronic stress	2.89 (5, 165)	.048	0.081 (0.003-0.144)	1.78 (1, 32)	.38	0.053 (0.000-0.244)
Arousal (momentary)	1.24 (5, 150)	.50	0.040 (0.000-0.085)	0.06 (1, 29)	.80	0.002 (0.000-0.118)
Valence (momentary)	2.13 (5, 150)	.17	0.066 (0.000-0.126)	0.13 (1, 29)	.81	0.005 (0.000-0.140)
Arousal (most recent stress event)	4.52 (5, 150)	.02	.131 (0.027-0.209)	0.11 (1, 29)	.81	0.004 (0.000-0.135)
Valence (most recent stress event)	1.96 (5, 150)	.19	0.061 (0.000-0.119)	0.44 (1, 29)	.73	0.15 (0.000-0.181)
SDRR^a^	1.62 (5, 110)	.26	0.069 (0.000-0.135)	1.13 (1, 21)	.50	0.051 (0.000-0.286)
RMSSD^b^	1.84 (5, 110)	.31	0.077 (0.000-0.148)	0.68 (1, 21)	.56	0.031 (0.000-0.253)
LF/HF^c^	3.91 (5, 109.01)	.03	0.152 (0.022-0.243)	0.32 (1, 20.99)	.70	0.015 (0.000-0.215)
Usability	24.80 (1, 45)	<.001	0.355 (0.138-0.522)	1.39 (1, 44)	.38	0.031 (0.000-0.177)
Satisfaction	7.12 (1, 45)	.05	0.137 (0.008-0.319)	4.03 (1, 44)	.12	0.084 (0.000-0.258)

a SDRR: SD of R-R intervals.

b RMSSD: square root of squared mean value of the sum of the differences of successive R-R intervals.

c LF/HF: quotient of low versus high frequency.

### Group Differences (H_2_)

No significant group effects were identified.

### Usability and Satisfaction Explorations

With regard to the usability, an increase in positive feedback from the participants was observed between the middle and the end of the study (*F*_1,45_=24.80; *P*<.001; η²*_p_*=0.355). A tendency of an effect also emerged in the satisfaction data, which, however, showed a significant decrease between the two measurement points (*F*_1,45_=7.12; *P*=.05; η²*_p_*=0.137). Technical issues occurred during the study, which inevitably disrupted measurements, particularly with regard to the sensor data. These disruptions primarily affected the connection between the sensor and the app in the initial weeks of the study. Furthermore, there have also been reports of discomfort caused by the daily demands of using the app and the sensor. Regarding the latter, the battery life was criticized.

## Discussion

### Principal Findings

This pilot study on the mobile app for occupational stress management developed in the RELAX project, according to the JITAI concept, has taken on the first evaluation of this app. The study aimed to determine the preliminary effectiveness of the app in terms of stress reduction during the study period and whether the degree of personalization of the stress interventions modifies the influence of the app. In addition to the indications of impact, the study also sought to examine the usability of the app in order to assess its transfer to a real-world context. The study’s findings, overall, were rather mixed in all respects.

### Evidence of Stress Reduction

Hypothesis 1, which predicted a reduction in stress levels by the end of the study compared to the baseline, could only be proven for certain stress indicators. Specifically, the subjective chronic stress measured daily and the subjective arousal of the most recent stress event in the daily EMA entries demonstrated a reduction in stress between given previous weeks and subsequent weeks. The substantial reduction in these stress variables occurred in the fifth and sixth week after the study began. These partial results align with prior research on stress management through mHealth [11,23] and offer preliminary indications of the app architecture’s effectiveness for the pilot study.

The absence of effects on other subjective measures of stress may be explained by the nature of the variables collected. The stress markers collected at untriggered, that is, self-selected measurement points of EMA entries, could contain a bias, where participants deliberately choose the EMA time points when they are not stressed. In the recommended EMA paradigm, the temporal parameters of measurement are typically prespecified by the researcher [54]. However, in this study, the subjects were permitted to select the temporal parameters of their observations autonomously, thereby allowing for the bias. Conversely, the question regarding the intensity of the most recent stress event in these EMA surveys specifically asks about stress situations, thereby ensuring that similar negative moments are recorded in this category for direct comparison. In contrast, the actual momentary experiences at EMA entry time could offer a much wider range of possible experiences in the moment, which might not provide clear direction when aggregated.

As indicated by prior studies [55], the manner in which stress is assessed using EMA has been found to influence the frequency with which subjects disclose their stress levels. Consequently, using explicit items regarding the most recent stressful event has been shown to yield more dependable data concerning stress compared to momentary inputs that are not based on events. The same rationale can be applied to the valence measurements. At the time of EMA entry, the valence may have been without clear direction in the spectrum of potential valences. It is noteworthy that the negative valence of the most recent stress event has remained constant, despite a decline in arousal levels during the most recent stress moment. According to the findings of research conducted on mood, the arousal level is more volatile and susceptible to interventions than the valence level [56]. However, empirical studies on stress management with just-in-time interventions show that the valence of an affect can be influenced after only a few weeks [57,58], so this aspect requires further consideration.

The absence of change in work-related stress prior to and following the study necessitates an alternative explanation for the self-reports. One possibility is that the instrument used to assess these constructs, the VEDAS [51], was predominantly influenced by the content of specific work-related factors. The items in the questionnaire address specific scenarios that have been identified as potential triggers for stress, such as the pressure to take work home or to extend one’s working hours. These factors might not have been adequately addressed by the interventions within the short time frame of this study. Despite the high degree of personalization of the interventions, it is possible that there was an absence of an appropriate intervention for every possible scenario. For instance, interventions involving broadband techniques, such as different types of relaxation or skills training, may not be immediately effective for a person in a highly specific scenario. A preponderance of extant literature already recommends that factors such as the individual, the context, and the content must be considered equally in a potential intervention if that intervention is to be truly effective [59]. The decision tree, with predefined structures for individual, context, and content factors, restricts this aspect. In this context, the validity of an instrument such as the VEDAS should be reconsidered, as the person-centered approach to interventions and the situation-based approach to this instrument may not be sufficiently aligned.

The physiological indicators demonstrated a contrary effect to the expected subjective stress reduction. In this case, the only observed effect in the change of stress levels was manifested as an increase in the HRV parameter of the LF/HF. In the context of the relationship between subjective and physiological stress markers, it is essential to consider the complex interplay between these two parameter classes. A recent review of the correspondence between daily stress parameters at different levels demonstrated that only approximately half of the studies examined exhibited an association between the two indicator classes [60]. It is also conceivable that the duration of the study was insufficient to elicit a discernible effect on the markers of HRV, which are generally regarded as being relatively stable. Chronic stress, as has been previously discussed in analogous studies, may not have exerted a perceptible influence due to its inherent stability [19].

The validity of the LF/HF ratio as a stress marker is also debated in the literature [61]. When derived from real-life conditions in long-term measurements, such as from everyday wearables, it appears to be an unreliable indicator [62]. In this study, LF/HF was the only HRV parameter to show a significant change, despite expectations of corresponding changes across the multiple HRV indices [63]. Taken together, these observations suggest that the physiological recordings in this study should be interpreted with caution and that their interpretive value may be limited. In addition, the shortcomings of sensor measurement, which in some cases miss up to 75% of the possible data due to limited compliance or technical challenges, must be addressed. Comparable studies in the health care sector have shown adherence rates of more than 50% for the majority of subjects, with a maximum of 95% of data achieved relative to the total possible data in the study periods [64].

Although there were promising changes in selected subjective stress markers that could indicate an accumulated intervention-related effect in the later weeks of the study, the possibility must also be considered that, given the lack of significant changes in other parameters, the overall finding is negligible, and the app was not able to reduce stress as intended. The only two stress indicators that demonstrated a reduction during the study period had to be assessed retrospectively on a daily basis. The arousal of the most recent stress event included the query of a period of several hours before the entry, and the daily subjective chronic stress required the assessment of the entire past day with regard to various stress dimensions. Consequently, despite the implementation of the EMA method, recall bias could affect the validity of the results, as the experience in question could not be measured precisely in real time [65]. As Shiffman [54] previously demonstrated, recall bias includes the tendency to handle negative and positive experiences differently. Therefore, there may have been a tendency to recall prompted negative experiences in a biased manner, resulting in systematic results that may not reflect reality.

### Analysis of Personalization and Usability

In consideration of the degree of personalization according to H_2_, it was observed that both levels of personalization provided by the algorithm, high and low, contributed equally to the changes in stress levels or the absence of such changes. It is important to note again that the duration of the experiment may have been too short to fully manifest the potential of the high degree version of personalization. The necessity for the algorithm to undergo further training following the splitting into two groups may indicate that the difference in user experience was not yet substantial enough to be reflected in differences in the stress scores. The necessity of navigating the decision tree for each intervention, which serves to narrow down potentially suitable interventions on an individual basis, underscores the fact that the user had still a considerable degree of influence on the system at this particular stage of development. Consequently, the automatic selection of interventions by the app may have been marginalized. As an alternative possibility, the extensive personalization options provided by a selection of nearly 200 interventions, which may have already exceeded the threshold for a discernible effect, could have resulted in a ceiling effect, thereby preventing a potential group effect. Comparable research, which focused on an expanded range of potential interventions, has previously discussed the simplification of the app again, a discussion that occurred subsequent to their analysis of the degree of personalization and the corresponding results [19].

Comparable research so far shows that multilevel approaches to stress management, in which several levels of data are considered dynamically, show a high variability in effectiveness [23]. In this context, the persuasiveness of an intervention must always be considered [66] because this study does not show whether the participants performed the interventions at all and, if so, correctly, because they were unguided. Given the early stage of the research, it is to be expected that the desired effects cannot yet be clearly identified. At this stage of development, not only the effectiveness factors may not be fully developed, but the usability factors are also only explored and optimized in several iterations of evaluation studies. In the present case, this was an initial evaluation, which means that it will remain unclear what proportion of the pattern of results is actually attributable to effectiveness factors and what proportion to usability factors.

An increase in usability was observed over the course of the study, as shown by the self-report data between the study phases and at the end of the study. The adaptation of the app during the study period, particularly subsequent to the initial phase, in alignment with the expressed preferences of users, can be regarded as a notable improvement. Concurrently, there were indications of a decline in satisfaction at the trend level. This finding suggests a contradictory interpretation: while users initially perceived the system as more usable, their preference for it decreased over time. During the course of the study, personalization mechanisms were integrated into the background of the app to tailor interventions to individual stress levels. This was anticipated to improve the user experience and satisfaction. The potential for the daily use of the app to induce exhaustion or discomfort could offer an alternative explanation for the observed outcomes. The technical modifications implemented in the application may have promoted its use; nevertheless, the intrinsic characteristics of its daily use may have resulted in an escalation in discomfort over time. In the context of mHealth for stress management, the consideration of digital stress is imperative, as it can readily engender an effect that is contrary to the intended objective [47].

The adherence rates indicate an overall rather low level of engagement in the daily study activities. Conversely, the relatively high incidence of subjective data indicated the general willingness of the participants, suggesting that the low sensor rates are most likely attributable to technical barriers. Consequently, there is an ongoing need for technical enhancements to ensure a seamless user experience and optimize functioning. The absence of seamless functionality in a digital system can significantly compromise user experience, potentially impeding the system’s intended functionality [45]. Consequently, this study sought to illustrate the potential of the JITAI concept through the use of the developed app on a system yet to be optimized.

### Strengths and Limitations

The present evaluation of the RELAX app demonstrates the initial results of a mHealth approach to the practical implementation of the JITAI concept in the domain of occupational stress management. The application’s objective is to deliver interventions that are not only time efficient but also content focused, with the aim of optimally addressing the individual’s situation and needs. This approach builds on earlier methods that either limited themselves to the compilation of diagnostic data or offered generic, one-size-fits-all interventions [11-13]. A notable aspect of the study is the remarkably low rate of attrition, with only 4 participants (8% of the total sample) dropping out, which is particularly noteworthy given the intensive nature of the repeated daily EMAs and the wearable monitoring process that included nocturnal recordings. Dropout rates from other mHealth studies on chronic diseases exhibited comparatively higher percentages, ranging from 40% to 49% [67]. Despite the presence of incomplete data recordings, adherence remained consistent, underscoring the necessity for such interventions in employees’ daily lives.

The assessment of multiple levels of stress markers made it possible to compare them, whereas previous studies of comparable approaches have focused on only a few parameters [13,23]. This approach allowed for the identification of variability in the effects across dimensions, which, although not in the expected manner, may provide valuable insights from alternative perspectives. It is acknowledged that stress markers do not always align, particularly between physiological and subjective parameters [68]. The findings from this study lay the foundation for future research to dive deeper into this divergence and potentially link it to underlying factors within the specific mHealth context.

This study fulfills the quality requirements of a pilot study. However, it must therefore also be evaluated with the limited significance of such a study. The absence of a control group without intervention, as previously identified as a limitation in the study protocol [38], prevents clear attribution of all changes in stress parameters due to the intervention. The study design used here does not provide the internal validity required for statements on the effectiveness of the intervention. At this juncture, the outcomes of this study can only be interpreted as effects that occurred during the study period, which may provide an indication of effectiveness that must be validated with a controlled follow-up study.

Furthermore, as previously stated in the discussion, the study period was relatively brief, thereby limiting the opportunities for user intervention. The small sample size precluded the ability to draw definitive conclusions; thus, the findings should be considered exploratory in nature. This is particularly evident in the group randomization, where the sample sizes were further diminished. Following the implementation of technical stabilization updates to the application and the completion of its development phase, which will enable fully automated intervention selection, the evaluation should be expanded to include a more substantial sample size and an extended study duration.

Moreover, the employees’ daily experiences in their highly individual roles and in different industries are likely to have generated such a variable set of situations that the stress events within and between individuals call into question the comparability of the EMA data. The app’s capacity to adapt to each unique situation and individual user could be considered overly ambitious for the current stage of development, particularly in light of the absence of complete automation in intervention selection at the time of evaluation. Consequently, users retained a substantial degree of active involvement and control over the app’s functionality. To enhance the validity and generalizability of the findings, it is recommended that future evaluations use more mature app systems that prioritize the comparability of the sample and incorporate control groups.

### Conclusions

In summary, the study preliminarily examined the impact of an mHealth app developed in accordance with the JITAI concept. The findings were inconsistent, with only two subjective stress markers demonstrating the hypothesized stress reduction, while one physiological parameter exhibited a debatable increase. The validity of the attribution of the observed effects to the performance of the app is not without question. It is conceivable that these effects are, in fact, an artifact of a system that has not yet been fully developed, if the effects are at all attributable to the system. These findings give rise to questions concerning the extent of the app’s limited usability in its present developmental stage, such as the ease of use of the app system, as well as the integration of generally consistent daily mHealth use in the work context. Nevertheless, the study identified several promising starting points for the future development of a functional and effective app that can address occupational mental stress in an adaptive manner in terms of time and content.

## Supplementary material

10.2196/79642 Multimedia Appendix 1 User interface of the RELAX app in German, from left to right: dashboard, ecological momentary assessment questionnaire, first level of decision tree, and guided breathing intervention.

## Abbreviations

AI: artificial intelligence
CBT: cognitive behavioral therapy
EMA: ecological momentary assessment
HF: high frequency
HR: heart rate
HRV: heart rate variability
JITAI: just-in-time adaptive intervention
LF: low frequency
LMM: linear mixed model
mHealth: mobile health
VEDAS: Valencia Eustress-Distress Appraisal Scale
