# Supplementary figures and images for: Multimodal Personalized Mobile Health Just-in-Time Adaptive Intervention for Occupational Stress Management: Pilot Study
Source: JMIR Mhealth Uhealth. 2026 May 18;14:e79642. doi: 10.2196/79642 (PMC13183344; doi:10.2196/79642)

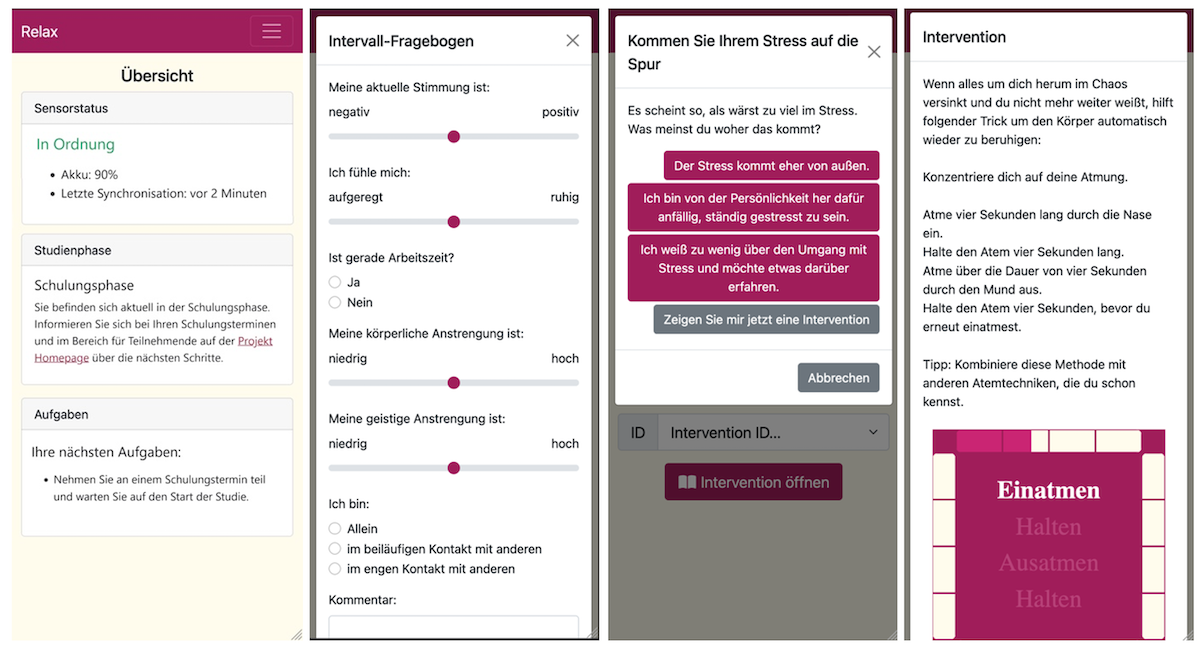

Supplement: Multimedia Appendix 1 [file mhealth-v14-e79642-s001.png]
